# Are Recent Medical Graduates More Skeptical of Vaccines?

**DOI:** 10.3390/vaccines1020154

**Published:** 2013-04-29

**Authors:** Michelle M. Hughes, Saad B. Omer, William K.Y. Pan, Ann Marie Navar-Boggan, Walter Orenstein, Edgar K. Marcuse, James Taylor, M. Patricia deHart, Terrell C. Carter, Anthony Damico, Neal Halsey, Daniel A. Salmon

**Affiliations:** 1 Department of International Health, Johns Hopkins Bloomberg School of Public Health, Baltimore, MD 21205, USA; E-Mails: mmergler@jhsph.edu (M.M.H.); somer@emory.edu (S.B.O.); amnavar@gmail.com (A.M.N.-B.); nhalsey@jhsph.edu (N.H.); 2 Hubert Department of Global Health, Rollins School of Public Health, Emory University, Atlanta, GA 30322, USA; E-Mail: worenst@emory.edu; 3 Nicholas School of Environment & Duke Global Health Institute, Duke University, Durham, NC 27708, USA; E-Mail: William.pan@duke.edu; 4 Duke University Medical Center, Durham, NC 27705, USA; 5 Emory University School of Medicine, Atlanta, GA 30322, USA; 6 Seattle Children’s Hospital & Department of Pediatrics, University of Washington, Seattle, WA 98105, USA; E-Mail: edgar.marcuse@seattlechildrens.org; 7 Child Health Institute, University of Washington, Seattle, WA 98115, USA; E-Mail: uncjat@u.washington.edu; 8 Office of Immunization and Child Profile, Washington State Department of Health, Olympia, WA 98504, USA; E-Mail: Pat.deHart@doh.wa.gov; 9 American Academy of Pediatrics, Elk Grove Village, IL 60007, USA; E-Mail: terrellcline@gmail.com; 10 Kaiser Family Foundation, Washington, DC 20005, USA; E-Mail: adamico@kff.org

**Keywords:** vaccines, health care surveys, health care provider/services

## Abstract

Rates of delay and refusal of recommended childhood vaccines are increasing in many U.S. communities. Children’s health care providers have a strong influence on parents’ knowledge, attitudes, and beliefs about vaccines. Provider attitudes towards immunizations vary and affect their immunization advocacy. One factor that may contribute to this variability is their familiarity with vaccine-preventable diseases and their sequelae. The purpose of this study was to investigate the association of health care provider year of graduation with vaccines and vaccine-preventable disease beliefs. We conducted a cross sectional survey in 2005 of primary care providers identified by parents of children whose children were fully vaccinated or exempt from one or more school immunization requirements. We examined the association of provider graduation cohort (5 years) with beliefs on immunization, disease susceptibility, disease severity, vaccine safety, and vaccine efficacy. Surveys were completed by 551 providers (84.3% response rate). More recent health care provider graduates had 15% decreased odds of believing vaccines are efficacious compared to graduates from a previous 5 year period; had lower odds of believing that many commonly used childhood vaccines were safe; and 3.7% of recent graduates believed that immunizations do more harm than good. Recent health care provider graduates have a perception of the risk-benefit balance of immunization, which differs from that of their older counterparts. This change has the potential to be reflected in their immunization advocacy and affect parental attitudes.

## 1. Introduction

Despite the unparalleled success in improving both individual and population health through vaccination, there is evidence of increasing delay or refusal of some or all childhood vaccines with safety concerns commonly cited as a contributing factor [1,2,3,4,5,6].

As vaccination programs succeed in achieving high coverage, the visibility of the disease itself is dramatically reduced [7]. As a result of lower disease prevalence, parents have more familiarity through both experience and media with real or perceived potential adverse events following immunizations than from vaccine preventable diseases. Parental benefit-risk assessments change as both cultural perception of the threat of disease and personal experience with disease decline and some parents come to see the risk of vaccines outweighing the benefits. As cohorts who have forgotten or not experienced vaccine-preventable disease come of childbearing age, their support of vaccines may be less than cohorts who grew up experiencing the effects of polio, measles, rubella, and other infectious diseases. Older mothers are more likely to have children with up-to-date immunizations compared with younger mothers [4,8]. Although there are a variety of individual, geographic, socioeconomic and other factors associated with vaccine uptake, there is evidence that maternal age (a potential proxy for changing parental perceptions) may be the most important factor determining whether a child is fully immunized. 

This “cohort effect” on vaccine related perceptions, may also affect health care providers’ vaccination beliefs and practices. Younger health care providers who have lived and trained in developed nations are likely to have had little or no personal experience with many vaccine preventable diseases and have been exposed to extensive public discussion of vaccine safety and alleged adverse events [9].

Since health care providers are the most frequently used and trusted source for vaccine information, it is important to investigate a potential cohort effect. If health care providers have concerns regarding the safety or importance of immunizations for both individual and public health then they may affect their immunization practice and the counseling provided to parents and patients [10,11,12,13,14,15].

We investigated the association of health care provider year of graduation from school with key beliefs about immunization and disease susceptibility, disease severity, vaccine safety, and vaccine efficacy.

## 2. Experimental Section

### 2.1. Design

In a previous study [10], 1630 parents of fully immunized children and 815 parents of children who were exempt from at least one school immunization requirement were mailed surveys in 2004 to examine factors associated with parental vaccine refusal. The children were enrolled in elementary schools in Colorado, Massachusetts, Missouri, and Washington. Parents identified 806 unique primary health care providers who cared for their children at 2 and/or 5–6 years of age. Contact information could not be found for 94 of these providers (8%). Surveys covering vaccine knowledge, attitudes, and beliefs were mailed in 2005 to 712 of these parent-identified providers [16]. The Committees on Human Research at Johns Hopkins University approved this study.

### 2.2. Survey

Providers answered questions on a 5-point Likert scale regarding vaccine-preventable disease susceptibility and severity and vaccine efficacy and safety (Table 1). Diseases queried included diphtheria, pertussis, tetanus, measles, mumps, rubella, polio, *Haemophilus influenzae* type b (Hib), varicella, hepatitis B, invasive pneumococcal disease and influenza. The survey also included questions about key immunization beliefs outlined in Table 1 with responses on a 5-point Likert scale and providers were asked, “In what year were you awarded your primary clinical degree?”

**Table 1:** Questions asked of providers for each of the diseases and vaccines listed.

Constructs for Provider Vaccine Beliefs		
Construct	Question	Disease/Vaccine
Disease Susceptibility	How likely do you think an unimmunized child in the United States is to get the following diseases during the next ten years? *(Response Options: Impossible, Not Likely, Somewhat likely, Likely, Very Likely)*	Polio Invasive Haemophilus influenzae type b (Hib) Varicella/chicken pox Hepatitis B Invasive Pneumococcal Disease Influenza
Disease Severity	If an 8-year old child got these diseases, how likely is the child to be seriously ill? *(Response Options: Not serious at all, Not very serious, Somewhat serious, Serious, Very Serious)*	
Vaccine Efficacy	How well do you think each of these vaccines prevents disease? (If the child completes the full recommended series) *(Response Options: Not protective at all, Not very protective, Somewhat protective, Protective, Very Protective)*	Polio (Inactivated/IPV) vaccine Haemophilus influenzae type B (Hib) conjugate vaccine Varicella/chicken pox vaccine Hepatitis B vaccine S. pneumoniae conjugate (Prevnar/PCV7) vaccine Influenza (inactivated) vaccine
Vaccine Safety	How safe do you think these vaccines are? *(Response Options: Very unsafe, Unsafe, Somewhat safe, Safe, Very safe)*	

### 2.3. Data Analysis

Beliefs regarding disease susceptibility, disease severity, vaccine safety, and vaccine efficacy were analyzed by individual diseases and vaccines and, from these, overall constructs were created. For each category, the responses were averaged across vaccines or diseases (each disease or vaccine weighted equally) to create four overall constructs on the same 5-point Likert-scale. These scores and values for key immunization beliefs were dichotomized 1 to <4 *vs.* ≥4. Responses of “don’t know” were counted as missing data and excluded from the analysis. Provider graduation year was categorized into 10 approximately five-year intervals: 1954–1959, 1960–1964, 1965–1969, 1970–1974, 1975–1979, 1980–1984, 1985–1989, 1990–1994, 1995–1999, 2000–2002. Due to low numbers in the boundary cohorts, the 1954–1959 cohort (eight health care providers) was combined with the 1960–1964 cohort and the 2000–2002 (two health care providers) was combined with the 1995–1999 cohort. We modeled graduation year in a multiple ways (continuous, quartiles, z-scores, decades, and 5-year intervals) with similar results.

Providers were also categorized by patient vaccination status to explore this variable as a potential confounder or effect modifier as the provider sample was not representative of U.S. medical providers. Providers were classified as “non-exempt” if the only parents who identified them were parents of fully vaccinated children. Providers were classified as “exempt” if the only parents who identified them were parents of children exempt from at least one immunization requirement. Mixed providers were identified by at least one parent of an exempt child and one parent of a fully vaccinated child.

Associations between provider graduation year and provider vaccine beliefs were explored using logistic regression. Provider vaccine beliefs were set as the dependent variable and provider graduation year interval as the independent variables to test for association between provider vaccine beliefs and graduation year. Analyses presented include the odds ratios adjusted by provider exemption status. Odds ratios are interpreted as the change in odds of a particular vaccine belief for each five-year increase in the year a provider graduated from clinical training (younger *vs.* older), adjusted for exemption status.

Results were considered to be statistically significant if the p-values were ≤0.05. All analyses were conducted using Stata, version 10.

## 3. Results and Discussion

### 3.1. Results

Of the 712 provider surveys sent, 44 did not reach the provider due to death, retirement, or a closed practice, and 14 were sent to a non-health care provider resulting in 654 received surveys. Of the received surveys 103 providers declined to participate resulting in 551 valid surveys for an overall response rate of 84.3%. Nine providers who did not provide clinical training graduation year were excluded from the analysis leaving 542 unique providers. The mean and median provider graduation year was 1982 (standard deviation: 9.2 years; range: 1954–2002). There were 380 non-exempt providers, 86 exempt, 74 mixed, and 2 unidentified. Health care providers primarily were medical doctors (MD) (86%) but also included osteopathic doctors (OD) (7%), naturopathic doctors (ND) 2%, nurse practitioners (NP) (3%), registered nurses (RN) (1%), and a licensed practical nurse (<1%).

**Figure 1:**
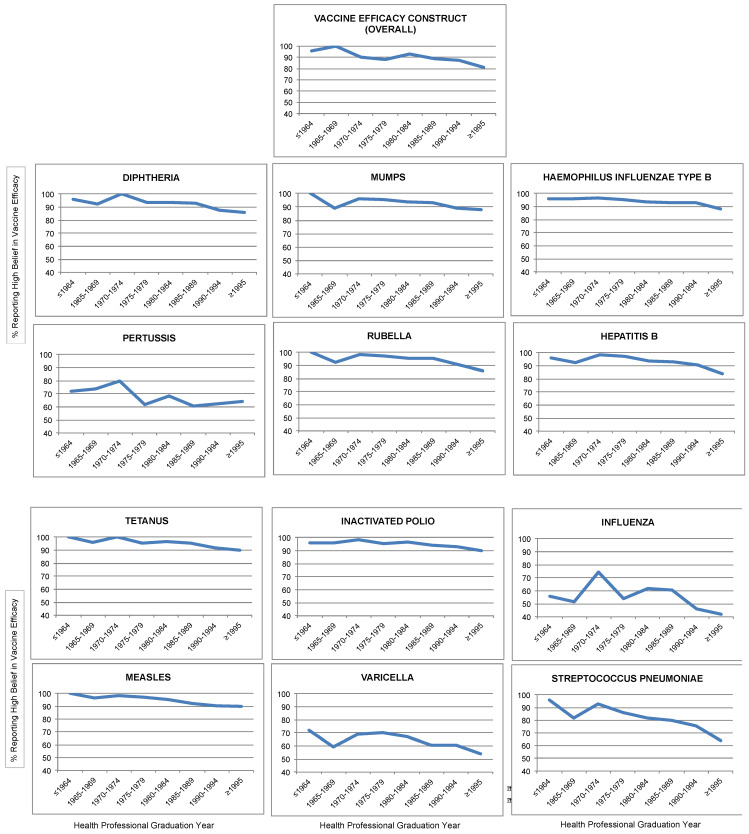
Percentage of providers reporting high belief in vaccine efficacy by year of health professional graduation.

**Figure 2:**
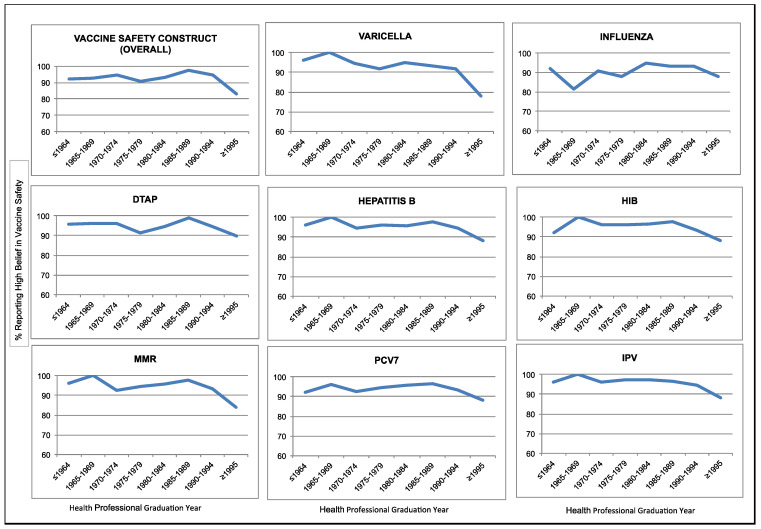
Percentage of providers reporting high belief in vaccine safety by year of health professional graduation.

Overall most health care providers had perceptions of high vaccine efficacy (89.4%) and high vaccine safety (92.7%). Lower percentages of providers believed in high disease susceptibility and severity (28.5% and 6.2%, respectively). Beginning with the cohort of providers graduating in the late 1980s there has been a slight downward trend among subsequent cohorts in beliefs in high vaccine safety (Figure 1) and high vaccine efficacy (Figure 2).

**Figure 3:**
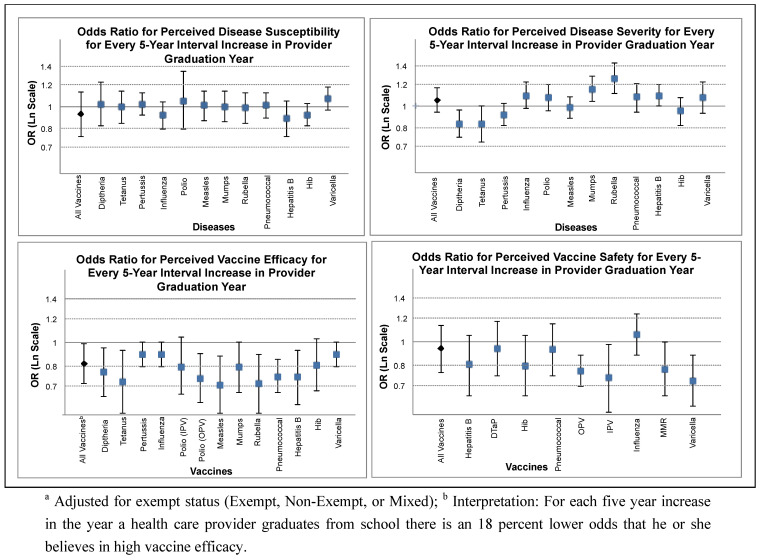
Odds ratio of perceived belief for every 5-year interval increase in provider graduation year.

Provider belief in overall vaccine efficacy was significantly associated with provider graduation year with graduates from a more recent 5-year interval having 15% decreased odds of believing vaccines are efficacious compared to providers from the preceding 5-year interval, adjusted for exemption status (OR: 0.85, 95% CI: 0.73–0.99) (Figure 3). 

Looking at each vaccine individually, there was not a strong effect on belief in vaccine safety by graduation year, although there was a trend towards younger providers having lower belief in vaccine safety. For both types of polio vaccines (IPV and OPV) there were significant decreased odds of believing the vaccine was safe for recent compared to older graduates. 

Provider belief in vaccine-preventable disease susceptibility was not associated with provider graduation year both overall (OR: 0.94, 95% CI: 0.78–1.13) and for specific diseases (Figure 3). For overall perceived disease severity there was also no association with provider graduation year (OR: 1.05, 95% CI: 0.95–1.17). The direction of association was not consistent among individual diseases. Diphtheria, tetanus, and pertussis were perceived as having lower severity for more recent compared to more experienced health care providers. Conversely, mumps and rubella were perceived as having higher severity for more recent *vs.* more senior health care providers. 

In the analysis of specific key immunization beliefs, overall, more recent graduates had greater odds of holding beliefs less favorable to immunization compared to more distant graduates (Table 2). For every 5-year increase in graduation year, there was 24% reduced odds of believing that immunizations are one of the safest forms of medicine ever developed. Recent graduates also had 1.4 times higher odds of believing that immunizations do more harm than good compared to older providers. The one exception to this trend was for the belief that vaccines strengthen the immune system. While the association was small, there was a statistically significant 10% increased odds that more recent graduates will believe that vaccines strengthen the immune system compared to more experienced graduates in the previous 5-year cohort. 

### 3.2. Discussion

In our study, a strong majority of health care providers believe that vaccines are highly safe and efficacious, similar to findings from other studies [17,18,19]. However, the proportion believing vaccines are safe and efficacious is lower for those clinicians who have graduated in recent decades. Only a small proportion of health care providers held beliefs of high disease severity and disease susceptibility, and a small minority (3.7%) of recent provider graduates believed that immunizations do more harm than good, likely reflecting the overall downward trend in prevalence of and mortality from vaccine preventable disease in the U.S. [17,18,19]. This changing perception of the risk-benefit balance of immunization may signal a critical change in immunization beliefs in the new generation of providers compared to their older counterparts. 

Previous studies have shown that parents and providers may be hesitant to vaccinate primarily due to safety concerns. Our original hypothesis was that vaccine safety beliefs would have the strongest association with provider graduation year [10,20,21]. However, vaccine efficacy beliefs exhibited the strongest association with graduation year followed by beliefs about vaccine safety. 

Our overall analysis showed this skepticism in more recent health care provider graduates for the majority of our key immunization beliefs. In the stratified analysis we saw the strongest effect of graduation year among the non-exempt providers, a subtle or non-effect among mixed providers and no association for exempt providers. One explanation for this finding is that providers who are caring for exempt children are fundamentally different than providers caring for fully vaccinated children. As a result, graduation year does not play a significant role because these providers have already come to a conclusion on the benefits and risks of immunizations.

**Table 2:** Relationship between provider graduation year and key immunization beliefs (Adjusted for Exemption Status).

Key Immunization Beliefs *(Response Options: Strongly disagree, Disagree, Neither agree or disagree, Agree, Strongly agree)*	Agree or Strongly Agree			
	n	%	OR ^a^	95% CI ^b^
Children should only be immunized against serious diseases	236	44.2	0.97	0.88–1.1
Children get more immunizations than are good for them	43	8.1	1.2	1.0–1.4
I am concerned a child’s immune system could be weakened by too many immunizations	32	6.0	1.02	0.83–1.3
I am more likely to trust immunizations that have been around for a while	373	69.5	0.98	0.89–1.1
Immunizations are one of the safest forms of medicine ever developed	431	81.2	**0.76 ^c,d^**	**0.67**–**0.87**
Immunizations are getting better and safer all of the time as a result of medical research	473	89.1	**0.84**	**0.71**–**0.98**
Vaccines strengthen the immune system	358	67.6	**1.1**	**1.0**–**1.2**
For the overall health of a child, it is better for them to develop immunity by getting sick than to get a vaccine	28	5.2	1.2	0.92–1.4
Healthy children do not need immunizations	18	3.4	1.0	0.79–1.3
Immunizations do more harm than good	21	3.9	**1.3**	**1.0**–**1.8**
I am opposed to school immunization requirements because they go against freedom of choice	32	6.0	**1.3**	**1.0**–**1.6**
I am opposed to school immunization requirements because parents know what is best for their children	13	2.4	1.2	0.86–1.6
School immunization requirements protect children against getting diseases from unimmunized children	474	88.4	0.91	0.78–1.1

^a^ Odds Ratio; ^b^ 95% Confidence Interval; ^c^ Interpretation: For each five year increase in the year a provider graduates from health professional school there is a 24 percent decreased odds that he or she believes that immunizations are one of the safest forms of medicine ever developed, adjusted for exemption status; ^d^ Statistically significant results indicated in bold.

Previous findings on a cohort effect among providers are mixed. Gust and colleagues showed no difference in proportion of physicians who recommend all vaccines by age category (≤36, 37–42, ≥42 years) [22]. However, by using provider age rather than graduation date differences in educational cohorts may have been blurred. Taylor *et al.* found no correlation between practice immunization rates for children at 8 and 19 months of age and percentage of practitioners born before 1950 but this is a rough division that is now likely outdated [23]. Koepke *et al.* grouped children by years their provider had been in practice (<11 *vs.* ≥11 years) and then looked at the percentage who were up-to-date on vaccines [24]. While not statistically significant, providers who had been in practice greater than 10 years had a higher percentage of children with up-to-date vaccinations compared to those who had been in practice for 10 years or less. The above studies demonstrated interesting trends towards association of provider age and medical cohort with vaccination beliefs, however the analysis done in these studies was not intended to focus specifically on a cohort effect. Our study adds to this body of literature by examining immunization beliefs for specific vaccines and diseases and by more specific provider graduation year intervals.

An interesting finding of the study was the differential effect of provider graduation year on perceptions of vaccine *vs.* disease constructs. While we found a consistent effect of graduation year on vaccine safety and efficacy beliefs, we found a smaller and more nuanced effect on disease susceptibility and severity beliefs. This may indicate that the changing assessment of vaccine benefit and risk by graduation cohort directly related to perceptions of the vaccines themselves rather than to a changing perception of the risk of severity of disease. Similarly it may indicate that with such a low burden of disease, the efficacy and real or perceived side effect of vaccines may be the most significant factors contributing to vaccination beliefs [25].

One policy implication of these findings is that provider’s knowledge of disease risk, complications, vaccine efficacy, and safety should be explored to understand if the new provider assessment reflects knowledge gaps or different weighting of the risks of disease and the benefits and risks of vaccines. Similarly parent’s perception of the risks of vaccine preventable disease relative to the other perceived health risks of their children warrants exploration.

In addition, the values of providers with respect to the balance between individual autonomy in medical decision making and protecting public health should be explored to fully understand the nuances in immunization related beliefs revealed by this study. Health care providers are one of the most trusted sources for vaccine information and if they have concerns about vaccines it may influence the vaccination status of their patients [10,22,26]. Continued efforts are need to ensure that health care providers fully understand the risks of vaccine preventable disease, the benefits and risks of immunization for the individual child and benefits of immunization to public health. 

A limitation of this study was that we did not have information on health care provider’s precise age but instead used health professional graduation year as a proxy for age. However, it is likely that providers within each 5-year graduation cohort will have roughly approximate ages compared to providers in other graduation cohorts. Moreover, the expected cohort effect is reflected more by healthcare experience and time period of health profession education than by the provider’s age. Another limitation was lack of sufficient sample size so non-significant findings may be the result of actual non-significance or of a lack of sample size to detect the true association. This study had a cross-sectional design and therefore we cannot assume causal directional relationships between provider’s graduation year and vaccine-related beliefs. However, it is unlikely that vaccine-related beliefs influence a provider’s graduation year. The provider survey was nested within a larger case-control study design and therefore the providers were not representative of all U.S. health care providers but rather provide a spectrum of providers caring for both vaccine exempt and non-exempt children. Although other data supports the overlap of attitudes and exemptions, we do not have data on differences in vaccination coverage by health care provider age and therefore more in-depth research is warranted. Further, we adjusted for provider exemption status based on categorization using a small subset of their patients. This limited provider patient sample size may provide an inaccurate representation of their entire practice. Finally, the study was conducted in 2005 so the association between provider vaccine beliefs and graduation year may have changed for younger health care providers. 

## 4. Conclusions

These findings demonstrated that the most salient difference between recently graduated *vs.* senior health care providers was that younger providers have lower belief in vaccine efficacy and safety although association was most pronounced for efficacy. More recently graduated health care providers also had higher odds compared to older providers of holding key immunization beliefs supporting the idea that vaccines do more harm than good. These results likely reflect both decreasing prevalence of vaccine preventable diseases and increasing awareness of vaccine adverse effects. Vaccine-related medical curricula should emphasize the importance, effectiveness, and safety of childhood immunizations. Further investigation is needed to determine the impact of this cohort effect on immunization rates overall.
